# The Montagues and the Capulets

**DOI:** 10.1002/cfg.442

**Published:** 2004-12

**Authors:** Carole Goble, Chris Wroe

**Affiliations:** The School of Computer Science The University of Manchester Oxford Road Manchester M13 9PL UK

## Abstract

Two households, both alike in dignity, In fair Genomics, where we lay our scene, (One, comforted by its logic's rigour, Claims ontology for the realm of pure, The other, with blessed scientist's vigour, Acts hastily on models that endure), From ancient grudge break to new mutiny, When ‘being’ drives a fly-man to blaspheme. From forth the fatal loins of these two foes, Researchers to unlock the book of life; Whole misadventured piteous overthrows, Can with their work bury their clans' strife. The fruitful passage of their GO-mark'd love, And the continuance of their studies sage, Which, united, yield ontologies undreamed-of, Is now the hour's traffic of our stage; The which if you with patient ears attend, What here shall miss, our toil shall strive to mend.


**Comparative and Functional Genomics**
*Comp Funct Genom* 2004; **5**: 623–632.
Published online in Wiley InterScience (www.interscience.wiley.com). **DOI:** 10.1002/cfg.442

**Conference Paper**

### The Montagues and the Capulets

Carole Goble* and Chris Wroe
*The School of Computer Science, The University of Manchester, Oxford Road, Manchester, M13 9PL, UK*

**Correspondence to:
Carole Goble, The School of Computer Science, The University of Manchester, Oxford Road, Manchester, M13 9PL, UK.
E-mail: carole.goble/c.wroe@manchester.ac.uk*

Received: 11 November 2004
Revised: 15 November 2004
Accepted: 16 November 2004

### Abstract

### Prologue

**Two households, both alike in dignity, In fair Genomics, where we lay our scene, (One, comforted by its logic's rigour, Claims ontology for the realm of pure, The other, with blessed scientist's vigour, Acts hastily on models that endure), From ancient grudge break to new mutiny, When 'being' drives a fly-man to blaspheme. From forth the fatal loins of these two foes, Researchers to unlock the book of life; Whole misadventured piteous overthrows, Can with their work bury their clans' strife. The fruitful passage of their GO-mark'd love, And the continuance of their studies sage, Which, united, yield ontologies undreamed-of, Is now the hour's traffic of our stage; The which if you with patient ears attend, What here shall miss, our toil shall strive to mend.** **Copyright © 2005 John Wiley & Sons, Ltd.**

### Stage notes

1. *This paper is a write-up of the opening plenary talk of the SOFG2 conference* (**http://www.sofg.org/meetings**). *Delegates throughout the rest of the meeting named themselves as Montagues and Capulets — which was revealing in itself.*
2. *For the sake of effect, we make sweeping generalizations.*

### We lay our scene

In recent years, ontologies have taken centre stage as their importance within life sciences grows. Interoperating resources, intelligent mining and sharing knowledge, be it by people or computer systems, requires a consistent shared understanding of what the information means. The life science community have an immediate and pressing need for controlled vocabularies if they are to successfully glue together and classify the numerous results populating their expanding collection of data resources. As a measure of the interest in the topic, over 700 people attended the opening paper of the ontology track at ISMB 2004 in Glasgow (Joslyn *et al.*, 2004) and over 60 were locked out of the room demanding entry.

The effective development of large ontologies, and their wide deployment, requires appropriate languages and mechanisms. We need *languages* that permit the formal and explicit specification of the meaning of terms, so that these meanings are machine-interpretable, can be unambiguously shared and can be used to computationally infer new knowledge. We also need *mechanisms* for ontology development, deployment and maintenance. Conveniently, the Computer Science/Artificial Intelligence communities work on knowledge representation techniques and technologies that should benefit the Life Scientist. Life Scientists, in turn, supply the Computer Scientists with practical, realistic problems as an ideal source of requirements, and provide a community of early adopters to pilot their solutions. However, despite the obvious mutual benefit, the two communities often find themselves in conflict, mostly due to misunderstandings of the motivations that lie behind the communities, a lack of awareness of the aspects of their own characters that frustrate the other and, perhaps, a failure to recognize that collaboration will mean compromise. It was ever thus. We have

Copyright © 2005 John Wiley & Sons, Ltd.

a roadmap to chart the rivalry and reconciliations between these two Houses (Shakespeare, 1596). We follow this to make explicit the characters of these two Houses (or three as it turns out), highlight some of the reasons for their quarrels, and identify opportunities for reconciliation that we hope will lead to a happy outcome, rather than a tragedy.

### The Houses of Genomics

Bioinformatics is already an interdisciplinary topic encompassing the many disciplines of the 'omics' — genomics, proteomics, metabolomics, transcriptomics — together with chemoinformatics, medical informatics, phenotypical observation, phylogeny, anatomy and so on. This mixing of disciplines is itself a challenge and, added to that, is the challenge of underpinning the bioinformatics by introducing Computer Scientists. In addition, the fields of ontology and knowledge management have their own communities. Thus, *in fair Genomics, where we lay our scene* there are a number of Houses. In fact there are three, rather than the traditional two — Computer Scientists, Life Scientists and Philosophers.

### The Montagues

*One, comforted by its logic's rigour/Claims ontology for the realm of pure*. This is the House of Computer Science, knowledge management and artificial intelligence (AI). Their interests lay in the logics and languages needed for the organization and representation of ontologies and knowledge bases that can support intelligent reasoning and logical inference. Theory is their strong point, with a traditional desire for orderliness, consistency, coherency and proof. They like their knowledge to be well behaved and have developed methodologies to build ontologies cleanly from the top down, from scratch, with good principles. Because they are developing techniques for all applications, their results are expected to be generic. They have example ontologies but, as this community typically is concerned with the mechanics of the ontology rather than its content, the examples are usually small and pathologically designed to test the boundaries of the expressiveness of languages or challenge reasoning engines. However, there are some examples of content efforts from this community. For example, Opencyc currently has around 60 000 terms describing 'common ideas' made publicly available from the 260 000 or more concepts of the Cyc ontology (**http://www.opencyc.org**). Despite the fact that much of the work is with application stakeholders, Montagues tend towards 'technology push', using the application as an experimental sand box during their pursuance of academic excellence. This House has been active for around four decades and during that time have developed a startling and confusing number of languages — CycL, KL-ONE, RDF, RDFS, OIL, DAML + OIL, OWL, RuleML, SWRL (Gómez-Pérez and Corcho, 2002; Horrocks *et al*., 2004) — and the tools to go with them — FaCT, RACER, OilEd, Protégé, Protégé-OWL, OntoBroker, Jena (Denny, 2004). Their e-commerce cousins have added to the mix with Topic Maps, UML, RosettaNet and ebXML. This activity has escalated in recent years, motivated by the Semantic Web vision, which has led this community to engage intensely with the standardization activities of the W3C.

The Semantic Web is an extension of the current Web, in which information is given well-defined meaning, better enabling computers and people to work in cooperation. In practice, this is exposing the meaning of Web resources by assertions in a common data model, Resource Description Framework (RDF), and the publication and sharing of consensually agreed ontologies in RDF Schema (RDFS) or Web Ontology Language (OWL), so that metadata can be shared and background knowledge can be declared. We use this semantic fabric to query, filter, integrate and aggregate the metadata, and reason over the metadata and ontologies to infer more metadata. To declare a measure of confidence in the assertions and inferences, we attribute trust to the metadata and proof to the inferences. The idea is to create a platform for automated, computational, sentient agents to operate over. Then these agents can dynamically discover and combine resources and applications on behalf of users, e.g. to book a medical appointment or make travel arrangements (Berners-Lee *et al*., 2001). As a consequence, the Montagues are currently found in the World Wide Web Consortium (W3C) Semantic Web and Semantic Web Services Activities. They have also had to become more tolerant of confusion as, unlike their traditional knowledge bases, the Web is messy — inconsistent

Copyright © 2005 John Wiley & Sons, Ltd. 

metadata, multiple and overlapping ontologies, and competing and conflicting logical claims that make reasoning tricky.

### The Capulets

*The other, with blessed scientist's vigour/Acts hastily on models that endure.* This is the House of the Life Scientists. The world of bioinformatics is of pragmatics and practice, with a strong application pull. Their motivating vision is one of well-structured controlled vocabularies for information sharing, classification and indexing. These are used to enhance accurate retrieval, create common standards for annotation and support the mediation between and interlinking of the contents of different databases. Capulets have been classifying animals since Aristotle and Linnaeus. Increasingly, ontologies are being used for applications other than annotation, such as data mining. Whereas the Montagues see knowledge representation as an end in itself, the Capulets see it as a means to an end, and that end is Science. Their operating timescale is immediate; they have a problem now and they are in a hurry. Thus, their approach is 'build it, use it, and fix it later'. Ontologies have typically been seeded from keyword lists or by small groups of highly motivated service providers/users. They have been put to use immediately, so there is no futile attempt to 'get it right first'. Consequently, methods for evolution and change have been present from the start. Drawing from their legacy of database curation practice, the Capulets have developed workable methodologies for consensually developing community-wide ontologies, supported by sophisticated infrastructure (Bada *et al.*, 2004). The ontologies are specific rather than generic, focusing on gene products, microarray experiments, sequences, anatomy, etc. At the time of writing, 39 were available from the Open Biological Ontologies website (**http://obo.sourceforge.net**), a gathering place of the community. Most are simple in their structure — graphs or taxonomies — but their coverage, relevance and take-up is significant and real. The favourite child of the Capulets is the Gene Ontology, an international effort of over 18 000 concepts with wide adoption that has made a significant impact (GO Consortium, 2000). They are keen on using standards and tools, but do not hesitate to construct their own if none of those available is appropriate. The Capulets inhabit an increasingly crowded landscape as life sciences move to systems biology. The medical informatics and healthcare community has many ontologies of its own and increasingly this world needs to be linked to that of genomics.

### The Philosophers

*One, comforted by its logic's rigour/Claims ontology for the realm of pure.* The Montagues have cousins. These are also firmly in the realm of theory, but whereas the Montagues concentrate on the representation of conceptual models of truth that aid an application, the Philosophers seek a single model of truth itself. Some even believe in one universal, unifying ontology. They build foundational ontologies such as SUO (the Standard Upper Ontology) or DOLCE (a Descriptive Ontology for Linguistic and Cognitive Engineering) (Lehmann, 2004), that contain concepts such as 'perdurant' (an entity that extends in time but is not wholly present at any one time) and 'endurant' (an entity that is wholly present through time). Their motivations are the theories of parts and wholes (i.e. mereology), essence and identity, dependence qualities, composition and constitution, participation and representation, which they claim should form the foundation of all ontologies. However, their contributions are helpful — they have developed methodologies and patterns that are relevant to Life Scientists, e.g. when modelling parts and wholes of biological structures. They are not usually, however, concerned with earthly pursuits such as tools, and take the high intellectual ground when it comes to academic scholarship. They are also notoriously argumentative, not given to building consensus, and have been arguing since Aristotle's time. Their operating timescale is 'as long as it takes'.

We have three Houses, as shown in Figure 1. Each speaks a different language, each has a different agenda, each plays a different role and each works to a different timescale. In some ways these differences are beneficially complementary, but they also sow the seeds of conflict.

### Duels

*From ancient grudge break to new mutiny/When 'being' drives a fly-man to blaspheme.* ISMB 1998

Copyright © 2005 John Wiley & Sons, Ltd. 

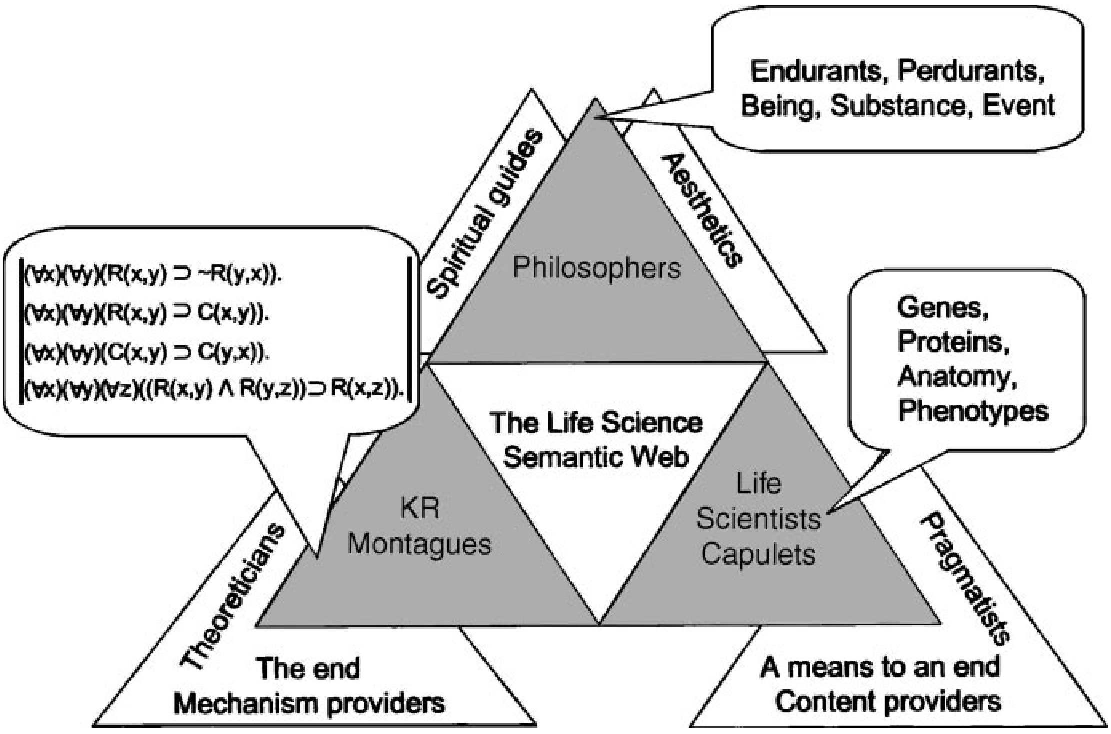

**Figure 1.** The three Houses of Genomics, showing their different characters, different languages and how they might be united through the Life Science Semantic Web hosted the first Bio-Ontologies workshop, which was an energetic affair. On the positive side, representatives of the Houses came together; on the negative side, the divisions between the hardcore members became apparent. Notably, a presentation of a foundational bio-ontology commencing with the concept 'being' led a breakaway group of Capulets, led by Professor Michael Ashburner, to found the Gene Ontology. Since then there have been other duels, usually started by the same old quarrels. As a service to genomics, here is a guide to the best ways to start an argument.

### How to frustrate a Capulet if you are a Montague

- *All or nothing*. Argue or imply that unless you are using all of the expressivity and reasoning capabilities of a knowledge representation language, e.g. OWL-DL (Horrocks *et al.*, 2003), then you shouldn't use it at all. A Montague often sees the language features but not the amount of effort needed to use them. The cost–benefit proposition for a particular application may not warrant such an effort; Wroe *et al.* (2003) outline the work needed to migrate from the current Gene Ontology to DAML + OIL. Using a fraction of the expressivity of a language and adopting a common exchange language has major benefits in itself (Stevens *et al.*, 2003).
- *Nine items or less*. Present an invented ontology example made up of 20 complicated artificial concepts, using every technical feature available, about wine or Clyde the elephant (a common toy example used in AI publications).
- *Suits me*. Offer an ontology tool that is straightforward if you have a background in logic but exposes the underlying formalisms in an unintelligible way to a domain scientist. This says, 'if you become like me, you too can use this' — which is at best unhelpful.
- *Keep still*. Present mechanisms that support creation of an ontology from scratch, but do not support changes or versioning of either the ontology itself, or the metadata that uses it. In fact, the response 'that's a research topic' to a predictable

Copyright © 2005 John Wiley & Sons, Ltd.

problem associated with an ontology as a consequence of it being living and working will be greeted with a sigh. Ontologies are conceptualizations of consensual knowledge of a community — the consensus changes, the communities evolve, and the conceptualizations change.

- *We know best*. 'Tell us what you want to say and we will build it for you'. Twenty years of work on knowledge elicitation and knowledge acquisition lead to a view that a 'subject matter expert' tells their knowledge to a knowledge engineer, who then encodes it. In the complex world of molecular biology research and related disciplines, where knowledge is the point of the scientific endeavour, this is at best a conceit. In the Halo project experiment (**http://www.projecthalo.com/**), analysis of the mistakes in the ontologies built showed them to be misunderstandings and simplifications by the knowledge engineers.
- *The finished product*. Refuse to release the ontology until it is 'finished', and when it is finished be astonished that the users won't use it. This is often because the ontology is designed in terms of the knowledge engineer, not the domain expert. Until surprisingly recently, the knowledge management community concentrated on the early parts of the knowledge life cycle, neglecting maintenance, in particular continued distributed development by a large number of knowledge contributors. Even today, many knowledge acquisition tools remain unconnected to ontology editors. Honourable exceptions exist, such as the GALEN tools for clinical terminology development — we observe that this was an application-driven project (Rogers *et al*., 1997). Protégé-OWL, part of the Co-ode project (**http://www.co-ode.org**), aims to eliminate the knowledge engineer middleman from the knowledge acquisition process and support domain experts to accurately and effectively build their own ontologies, drawing on the GALEN experiences.
- *Not my problem*. Offer to solve a different problem than the one actually presented. Maybe the problem can be dealt with by a sociological solution, which hardly ever attracts a Montague's interest but is feasible by well-organized communities of curators and knowledge contributors. Often a Montague would rather solve a harder problem (that to them is more interesting and fun), than take a simpler 'good-enough' route. At the heart of this lies the different agendas of the two Houses: Capulets want to link together scientific data well enough to get on with science, whereas Montagues want to build sentient applications.

### How to frustrate a Montague if you are a Capulet

There are two sides to every story:

- *Repeat the same old mistakes*. Make the same mistakes and the same misunderstandings, over and over again. Montagues have a wealth of experience in modelling, e.g. in mereology (Winston *et al*., 1987) and the differences between instances and concepts (Noy and McGuinness, 2001). They know that simple approaches using directed acyclic graphs do not gracefully scale. Manually predetermining and classifying every combination of every term is unnecessary and unsafe when logic languages automatically offer assistance (Rogers *et al*., 1998). Simplifications made early on in the development of an ontology, for understandable reasons, store up trouble down the road that can be foreseen if one is willing to pay the cost now rather than later. For example, combinatorial explosion of metabolic processes in the Gene Ontology eventually becomes difficult to maintain by hand and will lead to incompleteness in the structure and reduced performance in its intended database retrieval task (Wroe *et al*., 2003).
- *It works*. Hack together a mechanism, tool or application and declare it to work (with no evidence what that means) for a specific example, with no guarantee that it will work with any other data. Montagues are driven by generic solutions that are explainable, repeatable, sustainable and independent of freaks of data. They abhor baroque solutions with a large 'exceptions' case load.
- *Ignorance is bliss*. Ignore the past four decades of reasoning and knowledge representation research, along with the understanding of the boundaries of expressivity of languages and the algorithms that infer knowledge using them. A language with every construct in it, like OWL-Full, is not decidable. Error tolerance is not the same

Copyright © 2005 John Wiley & Sons, Ltd. 

as ignorance of errors and inconsistencies. Just as our understanding of the genome has advanced astonishingly over the past decade, so astonishing advances have been made in computing in understanding of the decidability and tractability of expressive knowledge languages.

- *I know what it means* — but that doesn't mean that anyone or anything else will. Machine-computable ontologies need formal and explicit semantics. A shared common understanding requires unambiguously specified clear semantics.
- *I tried your software and it broke/stopped responding, so I went off and built my own.* The majority of life science ontologies are large; the Gene Ontology currently stands at 18 000 concepts. These are much larger than the examples used to develop the Montagues' software, which in the past has struggled to cope. Scalability and performance do matter but are not usually the top priority for the Montagues.
- *Keep it simple.* A little semantics goes a long way; what a waste of the language when its richness is ignored! There is plenty of complexity to be had if it can be managed, but a Capulet will not make something complex if simple works well enough.
- *Consensus outweighs complexity.* The realization that no matter how simply structured or scruffy the ontology, what counts is if everyone uses it, e.g. the SOFG Anatomy Entry List (**http://www.sofg.org/sael/**) is a just a list of few hundred terms but enough to bind a community. Similarly, it doesn't matter how smart and sound and complete the ontology is, if no one uses it, it doesn't count. To date, it is hard to find a 'smart' ontology that has made it into widespread use. SNOMED-RT, and its successor SNOMED-CT, is smart on the inside for maintenance purposes (Spackman *et al.*, 1997), but goes through a process of semantic materialization — which turns it into graphs — to make it appear simple to healthcare applications and users.

### How to frustrate a Montague or a Capulet if you are a Philosopher

The Philosophers have centuries of modelling experience and thought but can comfortably start an interminable argument with all and any of the Houses. The issue comes down to *practical engagement* with those actually building the ontology.

- *Finger pointing from the sidelines.* Declare that the hard working ontology curators are not doing it right but do not tell them why, or give them any practical tools or guidance. Do not tell them directly, using their public curation policies and mechanisms, but instead make public statements or whisper in back rooms.
- *I wouldn't start from here.* Declare that the ontology should be started again, ignoring the effort needed and its extensive legacy, and offer no migration path for this legacy.
- *Mismatched expectations.* Complain that the ontology is not a model of true knowledge or does not fit a different purpose to the one for which it was developed. An ontology developed to annotate database entries is not intended to be a complete model of 'truth' and may well not suit an alternative application.
- *Truth and beauty.* Declare that 'truth' is more important than practicality. In reality, consensus is more important than truth and perfection is the enemy of the good. What is truth? German taxonomists considered the giant panda to be a bear; British taxonomists that it was a racoon; and American taxonomists didn't know which it was. Now it is defined as a bear — because the community has agreed on the definition of a bear and state it is a bear, not because it is the 'truth'.
- *There are no such things as concepts.* Take an esoteric stand that might win accolades in a journal of philosophy but is unhelpful in practice.

### Balcony scenes

*From forth the fatal loins of these two foes/Researchers to unlock the book of life;/Whole misadventured piteous overthrows/Can with their work bury their clans' strife.* Amid these arguments there are wonderful examples of reconciliation and mutual support. Many Capulets and Montagues are working closely together, and each complements the other, as we show in Figure 2.

### Using the W3C RDF/OWL standards

Both houses are in favour of standards — using them and creating them. The Montagues have

Copyright © 2005 John Wiley & Sons, Ltd. 

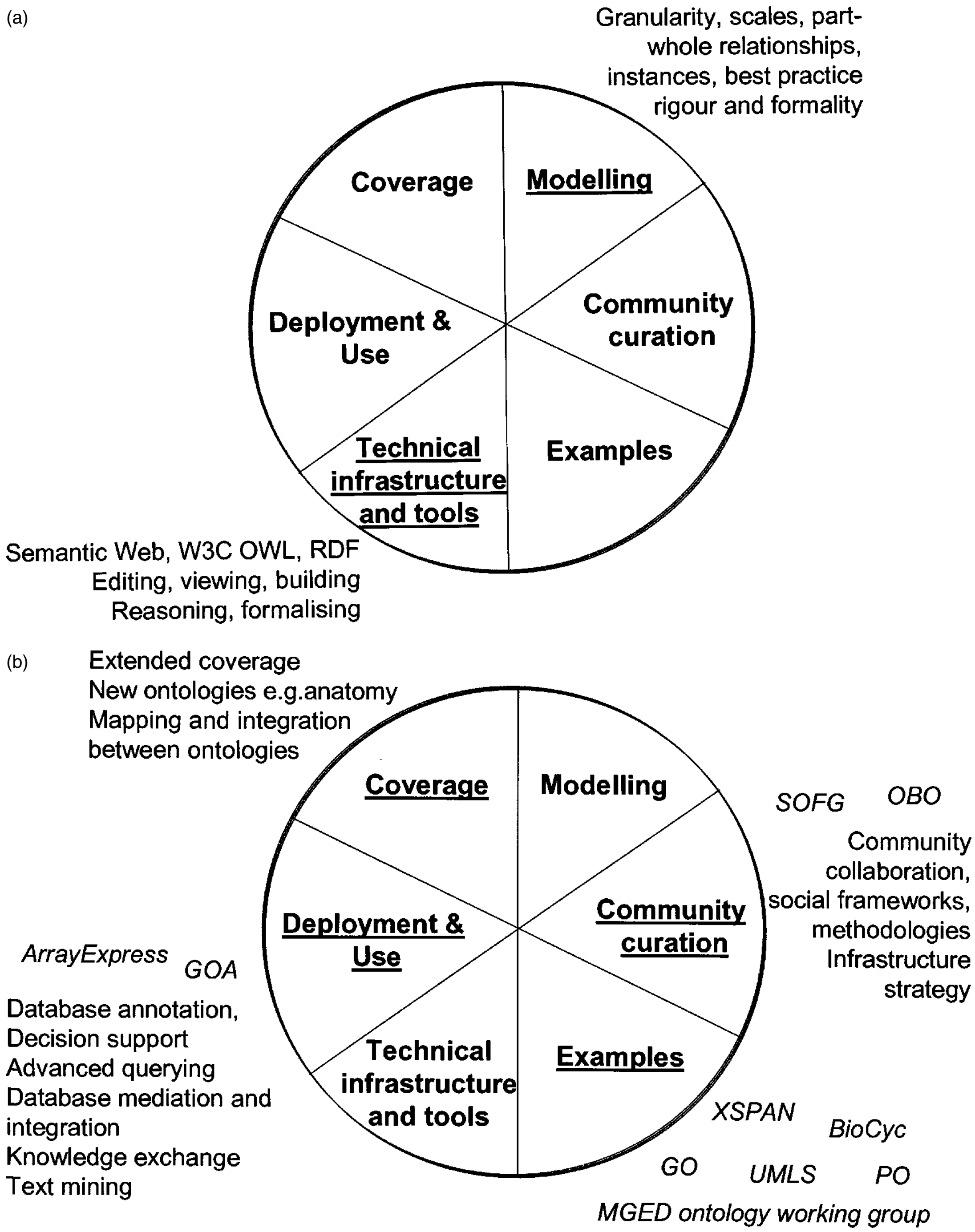

**Figure 2.** The current state of the art in bio-ontologies. (a) What the Montagues bring to the party. (b) The Capulets' contribution done a great job of producing a standard ontology language, OWL (Horrocks *et al.*, 2003), for the Semantic Web that draws from their years of research and practice. They have also produced the RDF language for describing assertions using ontology terms. The Capulets have proved to be enthusiastic early adopters of these languages. The Open Biological Ontologies (**http://obo.sourcefor-**

Copyright © 2005 John Wiley & Sons, Ltd. 

**ge.net/**) consortium mandates OWL as one of its preferred exchange languages; new ontologies, such as BioPAX (**http://www.biopax.org/**), are using OWL from the outset. Other work on the Gene Ontology uses language processing and patterns to extract implicit knowledge within it and uses reasoning to identify additional subsumption relationships and inconsistencies (Wroe *et al.*, 2003).

### The Age of Reasoning

Hand in hand with the adoption of OWL is the movement towards the judicial use of reasoning necessary to support the scale of the ontologies needed by the community. This includes figuring out when reasoning provides the best benefits in the ontology life cycle. Modelling using a compositional, term coordination approach (instead of pre-enumerating and classifying every term by hand) and a stronger emphasis on relationships between concepts motivates a need for reasoning, but reasoning is not the be-all and end-all.

### Tools

We see a convergence of ontology creation tools as the ontologies become more sophisticated, yet this complexity needs to be simplified for the user. OBO-Edit (**https://sourceforge.net/project/showfiles.php?group_id=36 855**) is becoming more like the full Protégé-OWL editor (Knublauch *et al.*, 2004), which in turn is adopting wizards and plug-ins to simplify and specialize interaction with the ontologies, like OBO-Edit. Tools like XSPAN (**http://www.xspan.org/**), developed for the life science community, can contribute more generally. The challenge is to lower the barriers of entry for developers and knowledge contributors, and provide 'invisible' tooling for end applications. We need tooling for vocabulary management and application developers, rather than core ontology development. We need scalable, efficient reasoning and RDF stores capable of dealing with millions of assertions.

### Ontology patterns

Presumably to the Philosophers' delight, the Capulets are turning to mereology for the more principled and systematic representation of taxonomies [e.g. in the Sequence Ontology (**http://song.sourceforge.net/**) and the Foundational Model of Anatomy (**http://sig.biostr.washington.edu/projects/fm/**)] and expanding to relationships other than is-a and part-of [e.g. in the Chemical Entities of Biological Interest dictionary (**http://www.ebi.ac.uk/chebi/**)]. Relationships in biology do not have the baggage that concepts have, and so represent a fresh opportunity for bio-ontologists.

### Methods

The Gene Ontology effort has created a gold standard method for community-wide consensual development of a working and living ontology (Bada *et al.*, 2004). Given that this works in practice, it is reassuring that more or less the same approach has now been proposed by the Montagues in the DILIGENT methodology (Pinto *et al.*, 2004), and thereby works in theory too. Incremental evolution is a day-to-day occurrence dealt with by highly curated ontologies like GO, and by such methodologies. Paradigm revolutions — where current scientific orthodoxy is overthrown, requiring a new ontology that is not an incremental extension of the old but is incompatible with the old — are less well supported, and a present a major technical and sociological challenge. If the effort to support paradigm shifts is too large we are in danger of fossilizing our knowledge. Experiences from the life sciences have shown that the success of large-scale ontology building is more sensitive to social or political processes than technology.

### Marriage or poison?

So our scene is laid. Can we help each other to shelter from the maelstrom of standards, languages, prototypes, tools, content and commercial offerings? Is a marriage between our Houses possible? One such bond is the Semantic Web initiative. It has motivated the Montagues to produce standard languages for ontologies and to tackle issues they had previously ignored, such as incomplete and inconsistent knowledge. The Capulets have already reaped the benefits. The Web needed incubation in a friendly, contained and forgiving community with a true distributed information problem (physics); the Semantic Web would benefit from the same and the ideal bedfellow is life sciences. In October 2004 W3C

Copyright © 2005 John Wiley & Sons, Ltd. 

hosted the first Semantic Web for Life Sciences meeting (**http://www.w3.org/2004/07/swls-ws.html**), which attracted over 100 delegates. Semantic Web technologies such as RDF and OWL, with domain-specific standards like LSID (Clark *et al.*, 2003), represent an evolvable, interoperable and fundamentally network-driven approach to information and knowledge aggregation. As such, they appear to represent an opportunity for developing solutions to overcome some of the difficult technology issues in life sciences — disparate, constantly evolving data sources and ontologies (in both public and private settings) and the need to aggregate the data and ontologies into a resource that can be queried, securely, and result in an audit trail.

However, this marriage could become poison for the Capulets if it is not entered into as an equal partnership. For example, during standardization process of OWL-DL, the expressivity to describe qualified number restrictions — the ability to say that a normal hand has five fingers and one of these must be a thumb — was abandoned. The technical know-how is well understood; it is logically expressible and tractable for reasoning. It isn't there because the W3C standards committee did not fully realize its obvious crucial importance to the life science community until it was too late, and the community did not voice its concerns clearly and loudly. The W3C Semantic Web Best Practice Activity has taken care not to make the same mistake. The Capulets must ensure that their interests are being served and that they are not merely an expedient test case for the Montagues. That means they must engage with them and their language design efforts, their standardization activities and their tool building.

*Romeo and Juliet* is a tragedy. How do we turn our story into a happy ending? Here is a desiderata for a good marriage. **Collaboration** through **Conversation**; set aside the time and patience to overcome the language barriers. Hold mutual **Respect** and **Understanding** for the other's motivations and contributions. Avoid being judgemental. **Compromise** should be viewed as a success and not a failure. Work as a **Partnership**. Take a look at each duel point and think about it. Do you say that? Do you hear that? How can we benefit from each other's experience and results?

As the Prince of Genomics might say (echoing the Prince of Verona; our peacekeepers are those who bridge all communities, typically originally trained in medical or life sciences, such as Alan Rector and Mark Musen):

*Rebellious subjects, enemies to peace*
*Throw down your mistemper'd weapons to the ground*
*Go hence, to have more talk of these top 'things';*
*All should be understood, respect'd, and well-found:*
*For never was a story with more GO*
*Than this of AI, life science and the O.*

### Acknowledgements

We would like to thank Robert Stevens, Chris Catton and William Shakespeare for ideas that contributed to this article and the SOFG talk that originated it.

### References

Bada M, Stevens R, Goble CA, *et al.* 2004. A short study on the success of the Gene Ontology. *J Web Semant* **1**: 235–240.

Berners-Lee T, Hendler J, Lassila O. 2001. The semantic web. *Scientific American* **May**.

BioPAX; **http://www.biopax.org/**

Chemical Entities of Biological Interest dictionary; **http://www.ebi.ac.uk/chebi/**.

Clark T, Martin S, Liefeld T. 2004. Globally distributed object identification for biological knowledgebases. *Brief Bioinform* **5**: 59–70.

CO-ODE; **http://www.co-ode.org**

Denny M. 2004. Ontology tools survey, revisited. O'Reilly xml.com; **http://www.xml.com/pub/a/2004/07/14/onto.html** (accessed 8 November 2004).

The Gene Ontology Consortium. 2000. Gene Ontology: tool for the unification of biology. *Nature Genet* **25**: 25–29.

Foundational Model of Anatomy; **http://sig.biostr.washington.edu/projects/fm/**

Gómez-Pérez A, Corcho Ó. 2002. Ontology specification languages for the semantic web. *IEEE Intell Syst* **17**: 54–60.

Halo; **http://www.projecthalo.com/**

Horrocks I, Patel-Schneider PF, van Harmelen F. 2003. From *SHIQ* and RDF to OWL: the making of a web ontology language. *J Web Semant* **1**: 7–26.

Joslyn CA, Mniszewski SM, Fulmer A, Heaton G. 2004. The Gene Ontology categorizer. *Bioinformatics* **4**(suppl 1): I169–I177.

Knublauch H, Fergerson R, Noy NF, *et al.* 2004. The Protégé-OWL plugin: an open development environment for semantic web applications. *Third International Semantic Web Conference.* ISWC2004 Lecture Notes in Computer Science, 3298. Springer-Verlag: Heidelberg 229–243.

Lehmann J, Borgo S, Masolo C, Gangemi A. 2004. Causality and causation in DOLCE. *Proceedings of the International Conference on Formal Ontology in Information Systems, FOIS 2004.* **114**: Frontiers in Artificial Intelligence and Applications.

MGED; **http://www.mged.org/**

Noy NF, McGuinness DL. 2001. Ontology Development 101: A guide to creating your first ontology. Stanford Medical

Copyright © 2005 John Wiley & Sons, Ltd. 

Informatics Technical Report SMI-2001-0880; **http://www-smi.stanford.edu/pubs/SMI_Reports/SMI-2001-0880.pdf** (accessed 8/11/2004).

OBO; **http://obo.sourceforge.net/**

OBO-Edit; **https://sourceforge.net/project/showfiles.php?group_id=36 855**

Opencyc; **http://www.opencyc.org**

Pinto HS, Staab S, Tempich C. 2004. DILIGENT: towards a fine-grained methodology for DIstributed, Loosely-controlled and evolvInG Engineering of oNTologies. European Conference on Artificial Intelligence, Valencia, Spain, 393–397.

Rogers JE, Price C, Rector AL, *et al*. 1998. Validating clinical terminology structures: integration and cross-validation of read thesaurus and GALEN. *Proc AMIA Symp* 845–849.

Rogers JE, Solomon WD, Rector AL, *et al*. 1997. Rubrics to dissections to GRAIL to classifications. *Stud Health Technol Inform* **43**(A): 241–245.

SOFG Anatomy Entry List; **http://www.sofg.org/sael/**

Semantic Web for Life Sciences; **http://www.w3.org/2004/07/swls-ws.html**

Sequence Ontology; **http://song.sourceforge.net/**

Spackman KA, Campbell KE, Côté RA. 1997. SNOMED-RT: a reference terminology for health care. *Proc AMIA Symp* 640–644.

Stevens R, Wroe C, Bechhofer S, *et al*. 2003. Building ontologies in DAML + OIL. *Comp Funct Genom* **4**: 133–141.

Shakespeare W. 1596. *Romeo and Juliet*.

Worldwide Web Consortium. 2004. W3C; Semantic Web Activity Statement; **http://w3c.org/2001/sw/**

Winston M, Chaffin R, Herrmann D. 1987. A taxonomy of part–whole relations. *Cogn Sci* **11**: 417–444.

Wroe CJ, Stevens R, Goble CA, Ashburner M. 2003. A methodology to migrate the Gene Ontology to a description logic environment using DAML + OIL. *Pac Symp Biocomput* **8**: 624–635.

XSPAN; **http://www.xspan.org/**

Copyright © 2005 John Wiley & Sons, Ltd. 
